# Wireless Fetal Heart Rate Monitoring in Inpatient Full-Term Pregnant Women: Testing Functionality and Acceptability

**DOI:** 10.1371/journal.pone.0117043

**Published:** 2015-01-26

**Authors:** Adeline A. Boatin, Blair Wylie, Ilona Goldfarb, Robin Azevedo, Elena Pittel, Courtney Ng, Jessica Haberer

**Affiliations:** 1 Department of Obstetrics and Gynecology, Massachusetts General Hospital, Harvard Medical School, Boston, Massachusetts, United States of America; 2 Division of Maternal Fetal Medicine, Department of Obstetrics and Gynecology, Massachusetts General Hospital, Harvard Medical School, Boston, Massachusetts, United States of America; 3 Department of Nursing, Massachusetts General Hospital, Boston, Massachusetts, United States of America, Boston, Massachusetts, United States of America; 4 Massachusetts General Hospital Center for Global Health, Harvard Medical School, Boston, Massachusetts, United States of America; Université de Montréal, CANADA

## Abstract

We tested functionality and acceptability of a wireless fetal monitoring prototype technology in pregnant women in an inpatient labor unit in the United States. Women with full-term singleton pregnancies and no evidence of active labor were asked to wear the prototype technology for 30 minutes. We assessed functionality by evaluating the ability to successfully monitor the fetal heartbeat for 30 minutes, transmit this data to Cloud storage and view the data on a web portal. Three obstetricians also rated fetal cardiotocographs on ease of readability. We assessed acceptability by administering closed and open-ended questions on perceived utility and likeability to pregnant women and clinicians interacting with the prototype technology. Thirty-two women were enrolled, 28 of whom (87.5%) successfully completed 30 minutes of fetal monitoring including transmission of cardiotocographs to the web portal. Four sessions though completed, were not successfully uploaded to the Cloud storage. Six non-study clinicians interacted with the prototype technology. The primary technical problem observed was a delay in data transmission between the prototype and the web portal, which ranged from 2 to 209 minutes. Delays were ascribed to Wi-Fi connectivity problems. Recorded cardiotocographs received a mean score of 4.2/5 (± 1.0) on ease of readability with an interclass correlation of 0.81(95%CI 0.45, 0.96). Both pregnant women and clinicians found the prototype technology likable (81.3% and 66.7% respectively), useful (96.9% and 66.7% respectively), and would either use it again or recommend its use to another pregnant woman (77.4% and 66.7% respectively). In this pilot study we found that this wireless fetal monitoring prototype technology has potential for use in a United States inpatient setting but would benefit from some technology changes. We found it to be acceptable to both pregnant women and clinicians. Further research is needed to assess feasibility of using this technology in busy inpatient settings.

## Introduction

Each year in the United States (US) approximately 4 million women give birth in hospitals, making childbirth the most common indication for admission to US hospitals [1]. Electronic fetal heart rate monitoring, first proposed in 1958, has become an integral part of inpatient labor care, with approximately 85% of all hospital births using this technology by 2002 [2]. Standard electronic fetal heart monitors have two main components—a Doppler ultrasound transducer used to detect the fetal heart rate and a tocodynamometer used to assess for uterine contractions. Both components are connected via cable to the patient and to a central non-portable unit equipped with a speaker and a printer. The fetal heart beat and its corresponding tracing, referred to as a fetal cardiotocograph, is then audible and visible to providers in the room and can be reviewed on a central monitor in real time when not in the room.

This system has been used successfully in both inpatient and outpatient settings at various gestational ages, stages of labor and with a variety of medical and obstetric conditions. It does however have some limitations. For antepartum fetal surveillance, such in-clinic or inpatient monitoring is time consuming and restricts access to those women able to reach facilities where fetal monitoring units are available. For pregnant patients in early or active labor these physical constraints severely restrict mobility and often subject a pregnant woman to bed-rest, whether desired or not, for the majority of her labor. Moreover the purchase and maintenance of this equipment comes with a hefty bill—approximately $1800 per delivery [3].

Advances in technology have overcome some of these limitations. Standard central units are now often equipped with an interface that stores and subsequently transmits the fetal cardiotocograms via modem allowing remote analysis of fetal cardiotocographs in real time either by central unit screens on inpatient labor wards or on computers equipped with specific software [4]. These systems still require pregnant women to present to health care facilities equipped with standard monitors. More recently, the use of a wireless fetal monitor, capable of transmitting fetal heart rate data via Bluetooth, was piloted amongst healthy patients undergoing labor induction at term and allowed home monitoring for 24 hours. Implementation and in-home use of wireless fetal monitoring technology as tested by that study was found to be feasible and acceptable to the pregnant women. [5].

The prototype technology under investigation has been studied in an outpatient setting where signal quality was found to be comparable to standard fetal monitors [6]. Extending wireless fetal monitoring to the inpatient setting, where the majority of births occur, requires additional investigation. Functionality may differ in a busy inpatient unit that often has multiple forms of monitoring and electronic equipment. Similarly acceptability may differ in pregnant women who are no longer in the comfort of their own homes. Furthermore, inpatient use of such devices requires buy-in and acceptance by clinicians who may be accustomed to standard monitoring. To date there are no studies assessing clinician views on wireless fetal monitoring.

In this pilot study we tested the technical function of a wireless fetal monitoring prototype technology in a tertiary care inpatient obstetric ward. We also assessed the perspectives of pregnant women and clinicians using the prototype in this setting.

## Methods

### Study design and participants

We performed a pilot cross-sectional, mixed methods study to test the functionality and acceptability of wireless fetal monitoring prototype technology in the inpatient obstetric ward of a single United States tertiary care hospital. Study participants were healthy, full-term pregnant women aged 18 and older, carrying singleton gestations, who presented to the Labor and Delivery Triage prior to the onset of active labor. Women were recruited as a convenience sample after the clinical reason for presentation was addressed by non-study clinicians and the women deemed to be clinically stable without evidence of active labor. Pregnant women and clinicians were recruited until no new data was obtained.

### Wireless fetal monitor prototype technology

**Fig 1:**
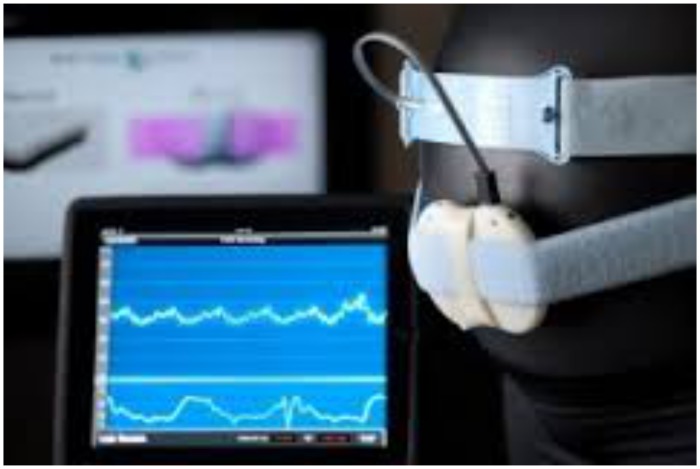
Wireless fetal monitoring prototype technology. Components illustrated include (from left to right): 1. Data gateway device (Android tablet) with fetal cardiotocograph output demonstrated on monitor. 2. Doppler monitor for assessing fetal heart beat attached with elastic strap to a model of a pregnant woman.

**Fig 2:**
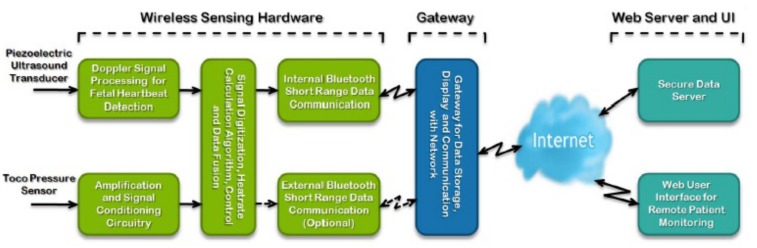
Components of the wireless fetal monitoring prototype technology [7] (from left to right): 1. Wireless Sensing Hardware represents the prototype technology as created by West Health Institute. This is capable of sensing fetal heartbeat and uterine contractions, digitizing the single, and transmitting this signal via Bluetooth technology. 2. The Gateway represents a smartphone or tablet device with operating system 4.2.1 or greater and capable of running a customized software application. The application on the device allows visualization of the fetal cardiotocograph in real-time, and submission of this data via the Internet to a web portal and user interface.

This study utilized a wireless fetal monitoring prototype technology developed by the Gary and Mary West Health Institute (WHI) (San Diego, CA) (See Fig. 1). Fig. 2 presents the architecture of the monitoring system [7]. The prototype technology is comprised of a non-invasive cardiotocograph that uses proven Doppler-based technology and sensors to measure fetal heart rate, uterine contractions and maternal heart rate. These components must connect (or “pair”) via Bluetooth to a data transmission gateway (i.e., a smartphone or tablet) on which the initial fetal cardiotocograph is visualized. Once fetal monitoring is complete data from the gateway device is sent in bulk via the Internet to a Cloud-based server for storage. Data is transmitted in encoded JavaScript Object Notation (JSON) with average file sizes of 200kb. Once uploaded, data can be reviewed on a customized web portal via password access from any device capable of web access. In our study we used a Samsung Galaxy Note (GT-N7000) for the data transmission gateway. The current prototype technology has been configured to work with Android devices (smartphone or tablet) with an operating system 4.2.1 or greater and capable of running customized software developed at the West Health Institute.

### Study procedures

After standard maternal and fetal monitoring was performed by non-study clinicians, consenting participants were asked to wear the prototype technology for 30 minutes. Two certified obstetric nurses qualified in fetal monitoring but with no prior experience with the prototype technology received training on the prototype in two separate sessions. Study nurses were then responsible for applying devices to patients, locating the fetal heart sounds, and transmitting data.

On review of data midway through the study we observed a delay between submission of a fetal cardiotocograph from the smartphone to the cloud-based server and availability of data for review on the web-portal. We then recorded the time data was submitted and the time it became available on website to quantify this delay and observe if changes instituted could reduce time delay.

Three obstetrician-gynecologists (authors AB, BW, IG) with experience in cardiotocography rated the fetal cardiotocographs obtained through the wireless fetal monitor for ease of readability. Cardiotocographs were rated on a scale of 1 to 5, 1 being difficult to read and 5, easy to read.

Following the completion of monitoring, study nurses administered closed-ended questionnaires to all pregnant women to assess acceptability. Five of these participants were asked to complete a longer in-depth interview with open-ended questions on likes and dislikes of the prototype compared with standard fetal monitoring. These participants were chosen based on interest and availability of time. Non-study clinical staff, available to participate, completed similar closed and open-ended questionnaires on acceptability.

### Data analysis

We defined a successful fetal monitoring session as recording a 30-minute fetal cardiotocograph, uploading this data to the cloud-based server, and being able to retrieve and review the cardiotocograph. Quantitative questionnaire data were summarized. A Wilcoxin rank sum test was used to compare data transmission times. We compared ease of readability scores among the three obstetricians by calculating an interclass correlation and 95% confidence interval. Open-ended responses were reviewed for common themes and summarized.

### Ethics statement

The Partners Healthcare Institutional Review Board approved this study, and all participants provided written, informed consent. All prototype technology was inspected and approved by the Biomedical Engineering Department at Massachusetts General Hospital.

## Results

A total of 38 participants were enrolled; 32 pregnant women wore the prototype technology and six clinicians observed their use. Baseline and demographic characteristics of participants are presented in Table 1.

**Table 1:** Characteristics of Study Participants.

Maternal Age at enrollment (yr)	33.8±5.0
>18–34 yr	19 (59.4%)
≥ 35 yr	13 (40.6%)
Race or ethnic group	
Black	1 (3.12%)
White (Non Hispanic)	22 (68.8%)
White (Hispanic)	3(9.3%)
Asian	3 (9.3%)
Other or unknown	3 (9.3%)
Parity	
0	17 (53.1%)
1	7 (21.9%)
2	8 (25%)
Gestational Age	39.6±1.0
Body-mass index at enrollment§	26.2±3.9
Education Level	
Some high School	2 (6.25%)
High School Diploma	3 (9.38%)
Some College	4 (12.5%)
College Graduate	9 (28.1%)
Graduate school	13 (40.6%)
Income Level	
<$50,000	8 (25.0%)
$50,000-$100,000	7 (21.9%)
>$100,000	13 (40.6%)
unknown/missing	4 (12.5)
Clinician Participant Characteristics	
Clinician Age at enrollment	34.7±9.5
>18–34 yr	3 (50%)
≥ 35 yr –	3 (50%)
Years in Clinical Practice at enrollment	9.5±5.8
Sex	
Female	6 (100%)
Male	0 (0)
Clinical Position	
Nurse	6 (100%)
Midwife	0 (0)
Doctor	0 (0)
Time spent interacting with devices	
1–15 minutes	1(16.7%)
16–30 minutes	5 (83.3%)

Numbers represent number (%, percent) or mean (± standard deviation) unless otherwise specified.

Race or ethnic group, education level and income level were self-reported.

§ Body mass index is the weight in kilograms divided by the square of the height in meters.

### Functionality

**Fig 3:**
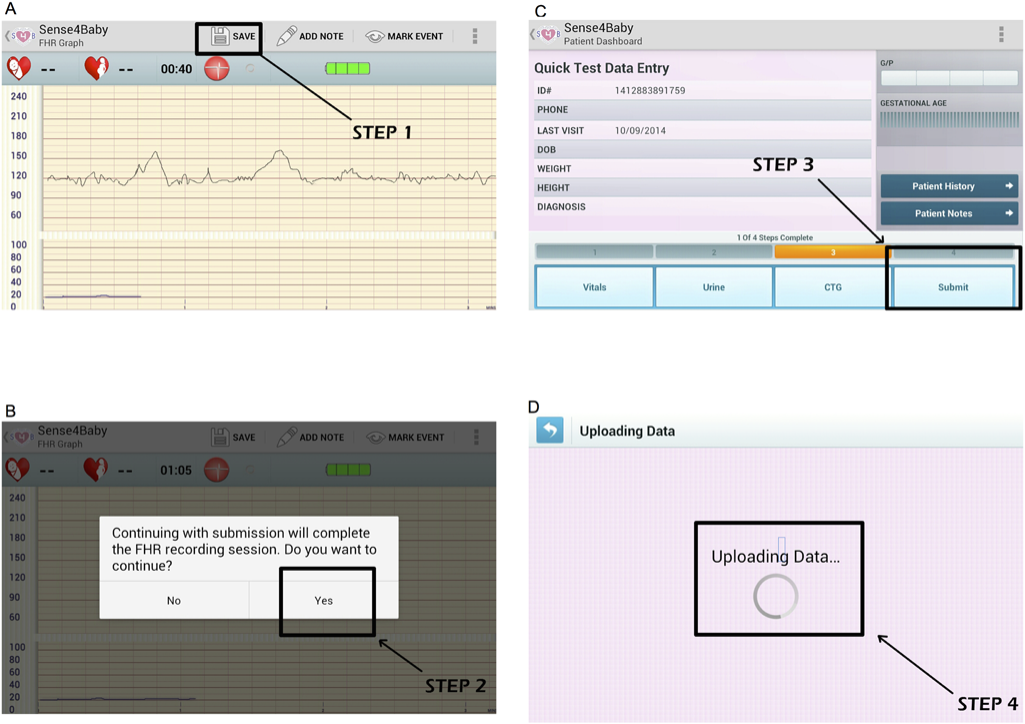
Illustration of steps required for submission of a completed monitoring session. In panel A, step 1 involves hitting the save button. In panel B, step 2 requires the user to confirm completion of the session. In panel C, the user must then hit “Submit “to complete step 3. In panel D, the screen with “Uploading Data” confirms submission is complete. In our experience step 3 was the most often missed step, as there is no prompt to hit the “Submit” button at this stage. This miss-step was correctable with further training and practice with the application.

We achieved successful fetal monitoring sessions in 28 of 32 (87.5%) of pregnant women. In all 32 women, the study nurses were able to locate the fetal heart rate with the ultrasound transducer and view the fetal cardiotocograph on the smartphone that was paired with the prototype technology. For four women, however, transmission from the smartphone to cloud storage failed as all 4 steps required at the smartphone interface where not completely understood by the study nurses (see Fig. 3). Further training on the interface prevented further data loss.

Timing of data transmission was recorded for 13 participants. The median time between submission and availability on the web-portal was 18 minutes (range of 2 to 209 minutes). Observed delays were found to be secondary to connectivity difficulties and high traffic on the available public hospital Wi-Fi system. Two back-up systems were introduced to counter the problem: first, a local hotspot was added to which the smartphones could wirelessly connect, and second, prepaid airtime was added to data-enabled SIM cards on the smartphones. The time for submission prior to these interventions (for 3 patients) was a mean and median of 100 minutes (range 2–209). Following the interventions, the duration decreased to a mean of 47 minutes and median of 18 minutes (range of 2–200 minutes). In two of these participants, a transmission time of 192 and 200 minutes was observed despite interventions when the smartphone had inadvertently been set to receive signal from hospital. All other transmission times were below 25 minutes. When comparing the transmission times relying on the hospital signal to those relying on the Smartphone, we found the change in times approached statistical significance, with a mean of 141 minutes for transmission relying on hospital Wi-Fi and a mean of 8 minutes for transmissions using the smartphone data-enabled SIM or local hotspot (p = 0.05).

**Fig 4:**
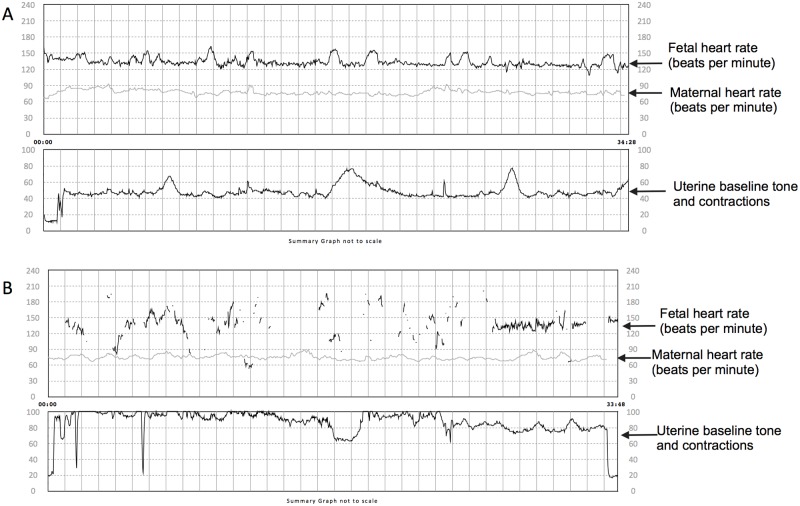
Examples of cardiotocographs obtained with the prototype technology. Panels A and B show (from top to bottom) fetal heart rate, maternal heart rate, uterine tone and contractions. The fetal cardiotocograph in panel A received a mean score of 5 on readability. The fetal heart rate depicted is continuous with no breaks and loss of contact allowing easy interpretation of the baseline heart rate and to assess for increases and decreases in heart rate. Panel B shows a fetal cardiotocograph that received a mean score of 1.7. Here lack of a continuous heart rate tracing prevents interpretation of the fetal heart rate at all times. For example it is hard to distinguish if gaps in the tracing represent decreases, increases or no change in the baseline fetal heart rate.

Ratings of ease of readability for the fetal cardiotocographs by the three obstetricians were high, with a mean of score of 4.3± SD 0.8, 4.3± SD 1.0 and 4.1± SD 1.2 respectively. The interclass correlation of these ratings was high at 0.81 (95%CI 0.45, 0.96), suggesting a high degree of agreement among the obstetricians. Fig. 4 illustrates an example of an easily readable tracing and a less readable tracing.

### Acceptability

Participant ratings of the wireless fetal monitoring are presented in Table 2. The majority of pregnant women found the prototype technology comfortable or very comfortable (65.6%). Most pregnant women and clinicians found it likeable or very likeable (81.3%, and 66.7%, respectively) and useful or very useful (96.9% and 66.7%, respectively). Most would either wear it again or have another patient wear it (77.4% and 66.7%, respectively).

**Table 2:** Acceptability of the wireless fetal monitor.

How comfortable did you find wearing the device?		
	Pregnant women	Clinicians
Very comfortable	12 (37.5)	n/a
Comfortable	9 (28.2)	n/a
Neutral/ok	11 (34.4)	n/a
Somewhat bothersome	0 (0)	n/a
Very bothersome	0 (0)	n/a
How useful did you find the device?		
Very Useful	15 (46.9)	4 (66.7)
Useful	16 (50.0)	0 (0)
Somewhat useful	1 (3.13)	2 (33.3)
Not at all useful	0 (0)	0(0)
How did you find the device?		
I really like it	11 (34.4)	2 (33.3)
I like it	15 (46.9)	2 (33.3)
Neutral/OK	6 (18.8)	2(33.3)
I do not like it	0 (0)	0 (0)
I really do not like it	0 (0)	0 (0)
Would you wear the device or have another patient wear it?		
I definitely would	25 (78.1)	4 (66.7)
I wouldn’t care one way or the other	7 (21.9)	2 (33.3)
I definitely would not	0(0)	0 (0)

Numbers indicate n (%).

### Participant reflections

Participants were generally positive about the prototype technology and identified several benefits of the wireless fetal monitoring devices; however, some concerns were noted. Representative quotations are presented from both pregnant women and clinicians below.

### Benefits of wireless compared to standard fetal monitoring

Most pregnant women reported that increased mobility was a desirable feature of wireless fetal monitoring and a reason for recommending the technology:

*“I think it’s a great idea especially since it allows for greater movement for mother-to-be.”*

*“Most people would like it…especially when you are not in active labor, being able to move around, that’s definitely a good thing”.*

*“… because you can move around with it and be monitored rather than having to be stuck in a bed, I think I would definitely recommend to others it that was an option to have the wireless”.*

Pregnant women also described the shape, material and size to be appropriate, stating, “it’s perfect, “I’m all for it”, and “maybe a bit smaller but fine all around”.

Clinician participants similarly appreciated the increased mobility, felt the prototype technology fit within clinical duties without problem, and would recommend it to other clinicians:

*“I think its great for the patients who are able to get up and move around. Also less cords to get tangled in.”*

*“It does not interfere with my clinical duties. I am still able to shave/prep and all other C-section preparation with it all. I am not worried about it harming my patient”.*

*“I would tell my colleagues that it is user friendly, convenient and safe… [they would find it] an intriguing idea.”*

Additionally, one clinician expressed the handheld prototype technology improved the monitoring experience:

*“It was interactive for the patient. It made her feel like she was involved in her care.”*

### Concerns

Despite generally positive feelings, two of the pregnant women expressed a theoretical safety concern:

*“I can also see people might be concerned about what kind of effect it does have on the baby or the mom…more of like radiation. I mean probably is just the words [description of the device] that sounds a little scary. It may not have any real impact, but when you talk about baby, everything is super cautious.”*

Clinicians identified concerns with patient education, including patient education, user interface challenges, and some design features:

*“Some clinicians might be concerned about patient education, that is, monitoring, watching their tracing”.*

*“… smaller screen [Smartphone] for older eyes.”*

“It was *too difficult to connect to hand held device”*

“It *could be annoying to be mobile with wire/cords hanging from the ultrasound and toco*”.

## Discussion

Our study results demonstrate the potential for clinician implementation of wireless fetal monitoring in a US inpatient obstetric setting; however further technology advances are needed to improve the functionality of the current prototype technology for the inpatient obstetric setting. Fetal cardiotocographs were scored highly on ease of readability with good consensus amongst three obstetricians. The majority of pregnant women and clinicians found the technology to be highly acceptable and desirable, although concerns regarding safety and design would need to be addressed for clinical implementation.

Strength and connectivity to a reliable Wi-Fi system represented the biggest challenge to timely transmission of data between the smartphone and the Internet. This problem reflects the finding in a previously reported study assessing wireless fetal monitoring, where technical difficulties with transmission were noted as the main reason for recall of remotely monitored patients [5]. File size was an unlikely contributor given the chosen format for transfer and the relatively small average file sizes for transmission (200kb). Additional we noted an improvement in transmission time using the non-hospital based Wi-Fi signal. Data transmission delay due to inadequate Wi-Fi signal is however an important lesson. Quality and availability of data infrastructure must be carefully established, even in “high tech” settings like a large, urban medical center in a high resource country. Fortunately, this problem was reduced, though not completely solved, using a separate Wi-Fi hotspot and using the smartphone internal cellular data system as a backup.

Delays in transmission also had a significant impact for the study obstetrician to review fetal cardiotocograms that had been submitted but not yet uploaded to the cloud. The flow of data in the current design of the wireless monitor and its interface does not allow for storage at the gateway stage preventing clinicians from reviewing fetal cardiotocograms in a timely fashion should any delays occur. This process could significantly impact clinical decision-making should a concerning fetal cardiotocogram be obtained and underscores the benefit of introducing the ability to store and visualize the fetal cardiotocograph on the gateway smartphone device. Clinicians at the bedside will then have immediate and constant access to the fetal cardiotocograph regardless of transmission. A data repository on the smartphone also has the advantage of expanding the use of the prototype technology to settings were Internet access is inconsistent or computers unavailable

With a range of up to 10 feet, the use of Bluetooth to transmit data from pregnant women to the gateway device could also limit the use of this prototype technology on a busy labor floor. This distance essentially ties the clinician wishing to observe the fetal cardiotocogram in real time to within 10 feet of the pregnant woman, thus essentially requiring a 1:1 ratio of nursing.

In both high and low resource settings, where labor floor nurses often have many competing tasks and may be in short supply, such requirements are likely impractical.

In summary we made the following conclusions and recommendations regarding the technical function of the wireless monitor and its use in an inpatient setting:

1. Interface complexity can lead to data loss.
  Study Recommendation:
  - Create simpler, “fool-proof” interfaces that require minimal to no training
  - Create a data repository on gateway device as back up in case of transmission failure
2. Wi-Fi connectivity may result in delays of data submission from gateway device to web portal.
  Study Recommendation:
  - Use in-built smartphone cellular data systems though this might not completely eliminate delay
  - Create data repository and interface to allow visualization of recorded cardiotocographs on the gateway device.
3. Bluetooth technology has limited range and therefore requires 1:1 nursing for close fetal heart technology
  Study Recommendation:
  - Consider alternative wireless technology for data exchange
  - Consider real time transmission of fetal cardiotocograph from gateway device to central monitor.

Our findings point to generally high acceptability. Pregnant women reported that the prototype technology was useful, comfortable, and likable. In particular our findings that women appreciate the freedom and mobility offered by wireless fetal monitoring are similar to those reported by Rauf et al in their 2011 study assessing remote fetal monitoring during home induction of labor[5]. This suggests that when given the option, women would prefer having increased mobility and freedom in both the outpatient and inpatient setting and uptake of such a device would be high.

Perhaps not surprisingly, safety concerns were expressed in comments made by pregnant women, and clinicians mentioned the importance of patient education and training. These findings emphasize the need for careful education of patients and clinicians alike prior to the use of any new technology introduced in this population. They also point to a potential advantage of using a technology like Bluetooth that is widely present in general public use and therefore less likely to cause serious safety concerns.

A strength of this study is the inclusion of the clinician perspective on acceptability, which has not been previously published. Like the pregnant women, clinicians found the prototype technology to be likeable and would recommend it to other patients and their colleagues. In particular, our finding that clinicians perceived the device as useful, reflected both in comments and questionnaire results, is encouraging feedback as this measure has been demonstrated to be the most important predictor of use of new technology by clinicians[8].

Though this study provides encouraging pilot data for the use of wireless fetal monitoring, it has several limitations. A small sample size and a relatively homogenous study population—for the most part white, highly educated, high-income pregnant women and clinicians, prevent generalizability to more diverse populations in different health settings. Familiarity and comfort with smartphone technology may also be higher in a large urban city in a high resource setting. Further investigation will be needed to assess whether findings would be similar in more rural and diverse populations, both within and outside the United States. In addition, this study does not assess the utility of wireless fetal monitoring in preventing or mitigating poor fetal outcomes by capturing the fetal cardiotocograph. Controversy still remains over the utility and benefit of continuous intrapartum electronic fetal monitoring [9]. This urges careful consideration as we consider expanding the use of this technology to settings with limited electronic fetal monitoring. Prior to such implementation rigorous research will be needed to demonstrate clinical benefit in these new settings.

In sum, our study highlights the potential for wireless fetal monitoring in the inpatient setting of a large, urban tertiary health care facility, and demonstrates that such technology will be generally well received by the pregnant women and clinicians receiving and providing care in this setting. We observed solvable problems but found that with adequate clinician training this device was used successfully. The lack of real-time transmission and observed delays limit the use of the current prototype technology in an inpatient labor unit. In its current form however, outpatient case-uses where real-time transmission is not needed could be considered. In addition, the portability and self-contained features of this device allows the opportunity to expand fetal monitoring to settings without the infrastructure to support the bulky and more complex standard machines. These technological advances therefore not only have the ability to improve the user experience in current fetal monitoring settings, but also to potentially expand its use to resource—limited settings both within and outside the United States. Once functionality of the device has been improved, further studies assessing the impact of wireless fetal monitoring on management of labor are needed. Inclusion of costs will be critical in evaluating this approach for routine care.
